# Correction: Leaf vein patterning is regulated by the aperture of plasmodesmata intercellular channels

**DOI:** 10.1371/journal.pbio.3001869

**Published:** 2022-10-19

**Authors:** Nguyen Manh Linh, Enrico Scarpella

In the Acknowledgments, the name Przemek Prusinekiwcz should appear as: Przemek Prusinkiewicz.
