# Supplementary material for: Leaf vein patterning is regulated by the aperture of plasmodesmata intercellular channels
Source: PLoS Biol. 2022 Sep 27;20(9):e3001781. doi: 10.1371/journal.pbio.3001781 (PMC9514613; doi:10.1371/journal.pbio.3001781)
Supplement: S1 Table — (DOCX) [file pbio.3001781.s001.docx]

## S1 Table. Reproducibility of Expression and Pattern Features

| *Figure* | *Panel* | *No. Leaves With Displayed Features /*  *No. Analyzed Leaves* | *Assessed Expression or Pattern Features* |
| --- | --- | --- | --- |
| 1 | B | See S1 Data | Narrow I-shaped midvein and scalloped vein-network outline |
| 1 | C | See S1 Data | Narrow leaf and open vein-network outline |
| 1 | D | See S1 Data | Narrow leaf, open vein-network outline, and vein fragments and/or vascular clusters |
| 1 | E | See S1 Data | Lobed leaf, open vein-network outline, and vein fragments and/or  vascular clusters |
| 1 | G | See S1 Data | Free vein end as narrow as the rest of vein |
| 1 | H | See S1 Data | Free vein end terminating in vascular cluster |
| 1 | I | See S1 Data | Small vascular cluster |
| 1 | J | See S1 Data | Large, elongated vascular cluster |
| 1 | K | See S1 Data | Large, round vascular cluster |
| 2 | G | 20/20 | Absent (erGFP and YFP) |
| 2 | H | 20/20 | Midvein (erGFP). Whole primordium (YFP) |
| 2 | I | 19/19 | Midvein and first loop (erGFP). Whole primordium but weaker at primordium tip (YFP) |
| 2 | J | 21/21 | Midvein and first and second loops (erGFP). Mainly restricted to veins in top half of leaf and nearly ubiquitous in bottom half of leaf (YFP) |
| 2 | K | 16/16 | Midvein; first, second, and third loops; and minor veins (erGFP). Mainly restricted to veins in upper three-quarters of leaf and nearly ubiquitous in lower quarter of leaf (YFP) |
| 2 | L | 17/17 | Midvein; first, second, and third loops; and minor veins (erGFP). Mainly restricted to veins in whole leaf except for lowermost part, where also in surrounding tissues (YFP) |
| 2 | M | 28/28 | Midvein (erGFP). Whole primordium (YFP) |
| 2 | N | 16/20 | Midvein (erGFP). Whole leaf but weaker at leaf tip (YFP) |
| 2 | O | 15/19 | Midvein and first loop (erGFP). Mainly restricted to veins in top half of leaf and nearly ubiquitous in bottom half of leaf (YFP) |
| 2 | P | 19/19 | Midvein and first and second loops (erGFP). Mainly restricted to veins in whole leaf except for lowermost part, where also in surrounding tissues (YFP) |
| 2 | Q | 19/19 | Absent (erGFP and YFP) |
| 2 | R | 35/37 | Midvein (erGFP). Mainly restricted to midvein in top half of leaf and nearly ubiquitous in bottom half of leaf (YFP) |
| 2 | S | 28/34 | Midvein and first loop (erGFP). Mainly restricted to midvein and first loop (YFP) |
| 2 | T | 37/44 | Midvein, first and second loops, and minor veins (erGFP). Mainly restricted to midvein, first and second loops, and minor veins (YFP) |
| 2 | U | 49/69 | Segments of midvein and first loop (erGFP). Mainly restricted to whole midvein and first loop in top half of leaf (YFP) |
| 2 | V | 49/53 | Segments of midvein and first loop (erGFP). Mainly restricted to whole midvein and first loop (YFP) |
| 2 | W | 38/47 | Segments of midvein and first and second loops (erGFP). Mainly restricted to whole midvein and first and second loops (YFP) |
| 2 | X | 47/55 | Segments of midvein and first, second, and third loops (erGFP). Mainly restricted to whole midvein; first, second, and third loops; and minor veins (YFP) |
| 2 | Y | 27/31 | Midvein (erGFP). Mainly restricted to midvein in whole primordium except for lowermost part, where also in surrounding tissues (YFP) |
| 2 | Z | 38/42 | Midvein, closed or open first and second loops, and vein fragments (erGFP). Mainly restricted to veins in whole leaf except for lowermost part, where also in surrounding tissues (YFP) |
| 2 | AA | 50/61 | Midvein (erGFP). Whole primordium (YFP) |
| 2 | AB | 31/38 | Midvein, closed or open loops, and vein fragments and/or vascular clusters (erGFP). Whole leaf but heterogeneous (YFP) |
| 3 | A | 30/32 | Midvein and closed first and open second loops |
| 3 | B | 28/34 | Midvein and open first and second loops |
| 3 | C | 63/79 | Vein formed in response to IAA connects to midvein |
| 3 | D | 27/44 | Vein formed in response to IAA runs parallel to midvein |
| 3 | E | 20/22 | Midvein and closed first loop |
| 3 | F | 24/24 | Midvein |
| 3 | G | 22/25 | Vein formed in response to IAA connects to midvein |
| 3 | H | 10/56 | Vein formed in response to IAA connects to midvein by broad vascular zone |
| 3 | I | 13/15 | Restricted to veins in whole leaf (erGFP). Mainly restricted to veins in top half of leaf and nearly ubiquitous in bottom half of leaf (YFP) |
| 3 | J | 20/22 | Restricted to veins in whole leaf (erGFP). Mainly restricted to veins in whole leaf except for lowermost part, where also in surrounding tissues (YFP) |
| 3 | K | 24/27 | Restricted to veins in whole leaf (erGFP). Mainly restricted to veins in middle of leaf and nearly ubiquitous on side of leaf where IAA was applied (YFP) |
| 3 | L | 18/26 | Restricted to veins in whole leaf (erGFP). Mainly restricted to veins in whole leaf except for lowermost part, where also in surrounding tissues (YFP) |
| 4 | A | 24/26 | Midvein, first and second loops, and minor veins |
| 4 | B | 24/30 | Midvein, closed or open first and second loops, and vein fragments |
| 4 | C | 15/20 | Midvein, open first loops, and vascular clusters |
| 4 | D | 54/58 | More lateral-veins, running parallel to one another in middle of leaf to form wide midvein and joining distal veins at margin of leaf to form smooth vein-network outline |
| 4 | E | 38/38 | More lateral-veins, running parallel to one another in middle of leaf to form wide midvein and joining distal veins at margin of leaf to form scalloped vein-network outline or ending freely in the lamina |
| 4 | F | 13/16 | More lateral-veins, running parallel to one another in middle of leaf to form wide midvein and ending freely in the lamina |
| 4 | G | 37/37 | Midvein (erGFP). Whole primordium (YFP) |
| 4 | H | 36/36 | Midvein (erGFP). Whole primordium (YFP) |
| 4 | I | 21/26 | Midvein, first and second loops, and minor veins (erGFP). Mainly restricted to veins in top half of leaf and nearly ubiquitous in bottom half of leaf (YFP) |
| 4 | J | 25/26 | Restricted to veins (erGFP). Throughout leaf but weaker along margin in top half of leaf (YFP) |
| 4 | K | 28/29 | Restricted to veins (erGFP). Mainly restricted to veins in whole leaf except for lowermost part, where also in surrounding tissues (YFP) |
| 4 | L | 27/27 | Whole marginal epidermis; all inner cells but stronger in midvein |
| 4 | M | 21/21 | Whole marginal epidermis; all inner cells but stronger in midvein and first loop |
| 4 | N | 28/30 | Whole marginal epidermis but weaker at leaf tip; in inner tissue, mainly restricted to midvein, first and second loops, and surrounding cells |
| 4 | O | 28/32 | Marginal epidermis in bottom half of leaf; in inner tissue, mainly restricted to midvein, loops, minor veins, and — in bottom half of leaf — surrounding cells |
| 4 | P | 30/30 | Whole marginal epidermis; all inner cells but stronger in midvein |
| 4 | Q | 37/40 | Whole marginal epidermis; all inner cells but stronger in midvein and continuous and connected first loop |
| 4 | R | 24/39 | Whole marginal epidermis; all inner cells but stronger, though heterogeneously so, in continuous and connected first loop |
| 4 | S | 36/61 | Marginal epidermis in bottom half of leaf; in inner tissue, mainly restricted to midvein, open first loop, and continuous and connected second loop and surrounding cells |
| 4 | T | 20/61 | Marginal epidermis in bottom half of leaf; in inner tissue, mainly restricted to midvein, open first loop, vein fragments and/or vascular clusters, and continuous and connected second loop and surrounding cells |
| 4 | U | 34/68 | Marginal epidermis in lower third of leaf; in inner tissue, mainly restricted to midvein, open first loop, and minor veins |
| 4 | V | 31/68 | Marginal epidermis in lower third of leaf; in inner tissue, mainly restricted to midvein, open first loop, minor veins, and vein fragments and/or vascular clusters |
| 4 | W | 39/61 | All inner cells in bottom half of leaf but stronger, though heterogeneously so, in continuous and connected second loop |
| 4 | X | 50/68 | In bottom half of leaf, mainly restricted to open second loop and minor veins and surrounding cells |
| 4 | Y | 19/20 | Whole marginal epidermis; all inner cells but stronger in midvein |
| 4 | Z | 18/22 | Whole marginal epidermis; all inner cells but stronger in midvein and continuous and connected first loop |
| 4 | AA | 14/22 | Whole marginal epidermis; most inner cells but stronger, though heterogeneously so, in continuous and connected first loop |
| 4 | AB | 24/32 | Whole marginal epidermis; in inner tissue, mainly restricted to continuous and connected first loop, though more heterogeneously so, and surrounding cells |
| 4 | AC | 25/32 | Marginal epidermis in bottom half of leaf; in inner tissue, mainly restricted to midvein, open loops, and vein fragments and/or vascular clusters |
| 4 | AD | 25/25 | Localized to plasma-membrane side facing veins to which second loop is connected |
| 4 | AE | 17/20 | Localized to plasma-membrane side facing contiguous cell in vein fragment |
| 4 | AF | 11/12 | Localized to plasma membrane side facing other cell in two-cell vascular clusters |
| 4 | AG | 27/27 | Localized to plasma membrane sides facing contiguous cells in larger vascular clusters |
| 5 | A | 21/23 | Midvein, first and second loops, and minor veins |
| 5 | B | 20/23 | Midvein, first and second loops, and minor veins |
| 5 | C | 22/25 | Midvein and open first loop |
| 5 | D | 20/20 | Midvein, open or closed first loop, and vein fragments |
| 5 | E | 27/30 | Midvein, open first loop, vein fragments, and vascular clusters |
| 5 | F | 20/26 | Midvein and scattered vascular clusters |
| 5 | G | 18/22 | Midvein, open first loop, and vascular clusters |
| 5 | H | 9/22 | Midvein, vein fragments, and vascular clusters |
| 5 | I | 9/22 | Midvein |
| 5 | J | 15/46 | Midvein, vein fragments, and vascular clusters |
| 5 | K | 27/46 | Midvein |
| 5 | L | 24/24 | Midvein (erGFP). Whole primordium (YFP) |
| 5 | M | 22/22 | Midvein (erGFP). Whole primordium (YFP) |
| 5 | N | 19/20 | Midvein, first and second loops, and minor veins (erGFP). Mainly restricted to veins in top half of leaf and nearly ubiquitous in bottom half of leaf (YFP) |
| 5 | O | 18/21 | Midvein and first loop (erGFP). Mainly restricted to veins in upper three-quarters and nearly ubiquitous in lower quarter of leaf (YFP) |
| 5 | P | 40/42 | Strong and mainly associated with veins |
| 5 | Q | 58/58 | Weak and broad |
| 5 | R | 26/31 | Strong and mainly associated with veins |
| 5 | S | 31/44 | Weak and broad |
| 6 | A | Se S3 Data | Narrow I-shaped midvein and scalloped vein-network outline |
| 6 | B | Se S3 Data | Open vein-network outline or narrow leaf and open vein-network outline |
| 6 | C | Se S3 Data | Lobed leaf, open vein-network outline, and vein fragments and/or  vascular clusters |
| 6 | D | Se S3 Data | Wide midvein and shapeless vascular cluster |
| 6 | E | Se S3 Data | Wide midvein and shapeless vascular cluster |
| 6 | F | Se S3 Data | Wide midvein and shapeless vascular cluster |
| 6 | H | 21/23 | Midvein, first and second loops, and minor veins |
| 6 | I | 25/29 | Wide midvein, dense network of thick veins, and thick vein-network outline |
| 6 | J | 22/42 | Wider midvein, denser network of thicker veins, and jagged vein-network outline |
| 6 | K | 20/42 | Even wider midvein, even denser network of even thicker veins, and pronouncedly jagged vein-network outline |
| 6 | L | 13/15 | Wide midvein and shapeless vascular cluster |
| 6 | M | 27/27 | Wide midvein and shapeless vascular cluster |
| 6 | N | 21/22 | Midvein (erGFP). Whole primordium (YFP) |
| 6 | O | 22/22 | Midvein, first and second loops, and minor veins (erGFP). Mainly restricted to veins in top half of leaf and nearly ubiquitous in bottom half of leaf (YFP) |
| 6 | P | 36/36 | Midvein (erGFP). Whole primordium but weaker in nonvascular tissues (YFP) |
| 6 | Q | 26/31 | Wide midvein and shapeless vascular cluster (erGFP). Mainly restricted to the wide midvein in the bottom half of the leaf; nearly ubiquitous in the top half of the leaf but weaker in nonvascular tissues (YFP) |
