# Supplementary material for: Leaf vein patterning is regulated by the aperture of plasmodesmata intercellular channels
Source: PLoS Biol. 2022 Sep 27;20(9):e3001781. doi: 10.1371/journal.pbio.3001781 (PMC9514613; doi:10.1371/journal.pbio.3001781)
Supplement: S2 Table — (DOCX) [file pbio.3001781.s002.docx]

## S2 Table. Origin and Nature of Lines

| *Line* | *Origin/Nature* |
| --- | --- |
| *cals3-2d* | (1); introgressed into Col-0 |
| *cals3-3d* | (1) |
| *gsl8-et2* | (2) |
| *gsl8-6* | SAIL_679_H10 (ABRC); (3, 4) |
| *gsl8-chor* | (5) |
| *gsl8-1* | SALK_111094 (ABRC); (6, 7) |
| *gsl8-2* | GK_851C04 (ABRC); (7, 8) |
| UAS::YFP | Transcriptional fusion of six copies of the UAS sequence (9) upstream of the -46 Cauliflower Mosaic Virus 35S promoter (10) to a translationally enhanced Venus-encoding sequence (11, 12) (primers: “TeVENUS Fwd XbaI” and “TeVENUS Rev SacI”) |
| E2331 | (13, 14) |
| Q0990 | (15, 16) |
| Q0950 | (15, 16) |
| J3281 | (15, 16) |
| J1701 | (15, 16) |
| PIN1::PIN1:YFP | (17) |
| PIN1::PIN1:GFP | (18) |
| DR5rev::nYFP^HS^ | (19, 20) |
| DR5rev::nYFP^ES^ | Transcriptional fusion of nine copies of the DR5rev sequence (21) upstream of the -46 Cauliflower Mosaic Virus 35S promoter (10) to EYFP-Nuc (Clontech) |
| *gn-13* | (6, 22) |

### References

1. Vaten A, Dettmer J, Wu S et al. Callose biosynthesis regulates symplastic trafficking during root development. Dev Cell. 2011;21:1144-1155.

2. De Storme N, De Schrijver J, Van Criekinge W, Wewer V, Dörmann P, Geelen D. GLUCAN SYNTHASE-LIKE8 and STEROL METHYLTRANSFERASE2 are required for ploidy consistency of the sexual reproduction system in Arabidopsis. Plant Cell. 2013;25:387-403.

3. Chen XY, Liu L, Lee E et al. The Arabidopsis callose synthase gene GSL8 is required for cytokinesis and cell patterning. Plant Physiol. 2009;150:105-113.

4. Sessions A, Burke E, Presting G et al. A high-throughput Arabidopsis reverse genetics system. Plant Cell. 2002;14:2985-2994.

5. Guseman JM, Lee JS, Bogenschutz NL et al. Dysregulation of cell-to-cell connectivity and stomatal patterning by loss-of-function mutation in Arabidopsis chorus (glucan synthase-like 8). Development. 2010;137:1731-1741.

6. Alonso JM, Stepanova AN, Leisse TJ et al. Genome-wide insertional mutagenesis of Arabidopsis thaliana. Science. 2003;301:653-657.

7. Töller A, Brownfield L, Neu C, Twell D, Schulze-Lefert P. Dual function of Arabidopsis glucan synthase-like genes GSL8 and GSL10 in male gametophyte development and plant growth. Plant J. 2008;54:911-923.

8. Kleinboelting N, Huep G, Kloetgen A, Viehoever P, Weisshaar B. GABI-Kat SimpleSearch: new features of the Arabidopsis thaliana T-DNA mutant database. Nucleic Acids Res. 2012;40:D1211-5.

9. Giniger E, Varnum SM, Ptashne M. Specific DNA binding of GAL4, a positive regulatory protein of yeast. Cell. 1985;40:767-774.

10. Odell JT, Nagy F, Chua NH. Identification of DNA sequences required for activity of the cauliflower mosaic virus 35S promoter. Nature. 1985;313:810-812.

11. Gallie DR, Sleat DE, Watts JW, Turner PC, Wilson TM. Mutational analysis of the tobacco mosaic virus 5’-leader for altered ability to enhance translation. Nucleic Acids Res. 1988;16:883-893.

12. Nagai T, Ibata K, Park ES, Kubota M, Mikoshiba K, Miyawaki A. A variant of yellow fluorescent protein with fast and efficient maturation for cell-biological applications. Nat Biotechnol. 2002;20:87-90.

13. Amalraj B, Govindaraju P, Krishna A et al. GAL4/GFP enhancer-trap lines for identification and manipulation of cells and tissues in developing Arabidopsis leaves. Developmental Dynamics. 2020;249:1127-1146.

14. Gillmor CS, Park MY, Smith MR, Pepitone R, Kerstetter RA, Poethig RS. The MED12-MED13 module of Mediator regulates the timing of embryo patterning in Arabidopsis. Development. 2010;137:113-122.

15. Haseloff J. GFP variants for multispectral imaging of living cells. Methods Cell Biol. 1999;58:139-151.

16. Sawchuk MG, Head P, Donner TJ, Scarpella E. Time-lapse imaging of Arabidopsis leaf development shows dynamic patterns of procambium formation. New Phytol. 2007;176:560-571.

17. Xu J, Hofhuis H, Heidstra R, Sauer M, Friml J, Scheres B. A molecular framework for plant regeneration. Science. 2006;311:385-388.

18. Benkova E, Michniewicz M, Sauer M et al. Local, efflux-dependent auxin gradients as a common module for plant organ formation. Cell. 2003;115:591-602.

19. Heisler MG, Ohno C, Das P et al. Patterns of Auxin Transport and Gene Expression during Primordium Development Revealed by Live Imaging of the Arabidopsis Inflorescence Meristem. Curr Biol. 2005;15:1899-1911.

20. Sawchuk MG, Edgar A, Scarpella E. Patterning of leaf vein networks by convergent auxin transport pathways. PLoS Genet. 2013;9:e1003294.

21. Ulmasov T, Murfett J, Hagen G, Guilfoyle TJ. Aux/IAA proteins repress expression of reporter genes containing natural and highly active synthetic auxin response elements. Plant Cell. 1997;9:1963-1971.

22. Verna C, Ravichandran SJ, Sawchuk MG, Linh NM, Scarpella E. Coordination of Tissue Cell Polarity by Auxin Transport and Signaling. eLife. 2019;8:e51061.
