# Supplementary material for: Leaf vein patterning is regulated by the aperture of plasmodesmata intercellular channels
Source: PLoS Biol. 2022 Sep 27;20(9):e3001781. doi: 10.1371/journal.pbio.3001781 (PMC9514613; doi:10.1371/journal.pbio.3001781)
Supplement: S3 Table — (DOCX) [file pbio.3001781.s003.docx]

## S3 Table. Genotyping Strategies

| *Line* | *Genotyping Strategy* |
| --- | --- |
| *cals3-2d* | “CALS3 FWD 1” and “CALS3 m REV2” |
| *cals3-3d* | “cals3-3d F” and “cals3-3d R”; *Taq*I |
| *gsl8-et2* | “ET2dCAPS F” and “ET2dCAPS R2”; *Hin*dIII |
| *gsl8-6* | *GSL8*: “SAIL_679_H10LP” and “SAIL_679_H10RP”; *gsl8-6*: “SAIL_679_H10RP” and “LBb1.3” |
| *gsl8-chor* | “chorus dCAPS F” and “chorus dCAPS R”; *Nla*IV |
| *gsl8-1* | *GSL8*: “GSL8 FWD” and “GSL8 REV”; *gsl8-1*: “GSL8 FWD” and “LBb1.3” |
| *gsl8-2* | *GSL8*: “GK_851C04LP” and “GK_851C04RP”; *gsl8-2*: “GK_851C04RP” and “o8474” |
| *gn-13* | *GN*: “SALK_045424 gn LP” and “SALK_045424 gn RP”; *gn*: “SALK_045424 gn RP” and “LBb1.3” |
