# Supplementary material for: Leaf vein patterning is regulated by the aperture of plasmodesmata intercellular channels
Source: PLoS Biol. 2022 Sep 27;20(9):e3001781. doi: 10.1371/journal.pbio.3001781 (PMC9514613; doi:10.1371/journal.pbio.3001781)
Supplement: S4 Table — (DOCX) [file pbio.3001781.s004.docx]

## S4 Table. Oligonucleotide Sequences

| *Name* | *Sequence (5ʹ to 3ʹ)* |
| --- | --- |
| CALS3 FWD 1 | ATCCCTTGTCAACTCAGG |
| CALS3 m REV2 | GAGAGATCTGAAGAGCTT |
| cals3-3d F | CCATCTCTTGTGCAACTTTACAATG |
| cals3-3d R | TATCAGGATCGAGAGGTAGGATATTATCG |
| ET2dCAPS F | AAATTGTATTGGATCGTGACCTGTAATCTTTCATGC |
| ET2dCAPS R2 | CTCCAATATCCTTCCTCTTAATGTTTGGATATAAGC |
| SAIL_679_H10LP | GCCCAGGTATACTAAGCTGGG |
| SAIL_679_H10RP | CTTTTTCTTCTAACGTGGGGG |
| LBb1.3 | ATTTTGCCGATTTCGGAAC |
| chorus dCAPS F | TCATGTGGATGCTTAGTGAACTGCTTCTTACTAACT |
| chorus dCAPS R | AGCCAACTGACCCAGTCTTCAAAATCCTCGAGGGTC |
| GSL8 FWD | TCACATGCATATAGCTGTGGG |
| GSL8 REV | TAGTTCCGCAGACAAAGTTGC |
| GK_851C04LP | TTCAGAAGTTGCATCTGCATG |
| GK_851C04RP | ACACTCTGGAAGAAAGCGGAC |
| o8474 | ATAATAACGCTGCGGACATCTACATTTT |
| TeVENUS Fwd XbaI | GCGCGCTCTAGAGTATTTTTACAACAATTACCAACAACAAC |
| TeVENUS Rev SacI | AAAGAGCTCTTACTCGTCCATGCCGAGAGTG |
| SALK_045424 gn LP | TGATCCAAATCACTGGGTTTC |
| SALK_045424 gn RP | AGCTGAAGATAGGGAATTCGC |
