# Supplementary material for: Leaf vein patterning is regulated by the aperture of plasmodesmata intercellular channels
Source: PLoS Biol. 2022 Sep 27;20(9):e3001781. doi: 10.1371/journal.pbio.3001781 (PMC9514613; doi:10.1371/journal.pbio.3001781)
Supplement: S5 Table — (DOCX) [file pbio.3001781.s005.docx]

## S5 Table. Confocal Light Paths

| *Fluorophore* | *Laser* | *Wavelength (nm)* | *Main Dichroic Beam Splitter* | *First Secondary Dichroic Beam Splitter* | *Second Secondary Dichroic Beam Splitter* | *Emission Filter (Detector)* |
| --- | --- | --- | --- | --- | --- | --- |
| Lignin | HeNe | 543 | HFT 405/488/543 | Mirror | NFT 515 | BP 600–650 (PMT3) |
| YFP; Autofluorescence | Ar | 514 | HFT 405/514/594 | NFT 595 | NFT 515 (PMT3); Plate (META) | BP 520-555 IR (PMT3); 593–754 (META) |
| GFP; YFP | Ar | 458; 514 | HFT 458/514 | NFT 595 | NFT 545 (PMT2); NFT 545 (PMT3) | BP 475–525 (PMT2); BP 520-555 IR (PMT3) |
| GFP; YFP; Autofluorescence | Ar | 458; 514 | HFT 458/514 | NFT 595 | NFT 545 (PMT2); NFT 545 (PMT3); Plate (META) | BP 475–525 (PMT2); BP 520-555 IR (PMT3); 657–754 (META) |
| GFP; Autofluorescence | Ar | 488 | HFT 405/488/594 | NFT 545 | NFT 490 (PMT3); Plate (META) | BP 505–530 (PMT3); 550–754 (META) |
| YFP | Ar | 514 | HFT 405/514/594 | NFT 595 | NFT 515 | BP 520-555 IR (PMT3) |
