# Supplementary figures and images for: Leaf vein patterning is regulated by the aperture of plasmodesmata intercellular channels
Source: PLoS Biol. 2022 Sep 27;20(9):e3001781. doi: 10.1371/journal.pbio.3001781 (PMC9514613; doi:10.1371/journal.pbio.3001781)

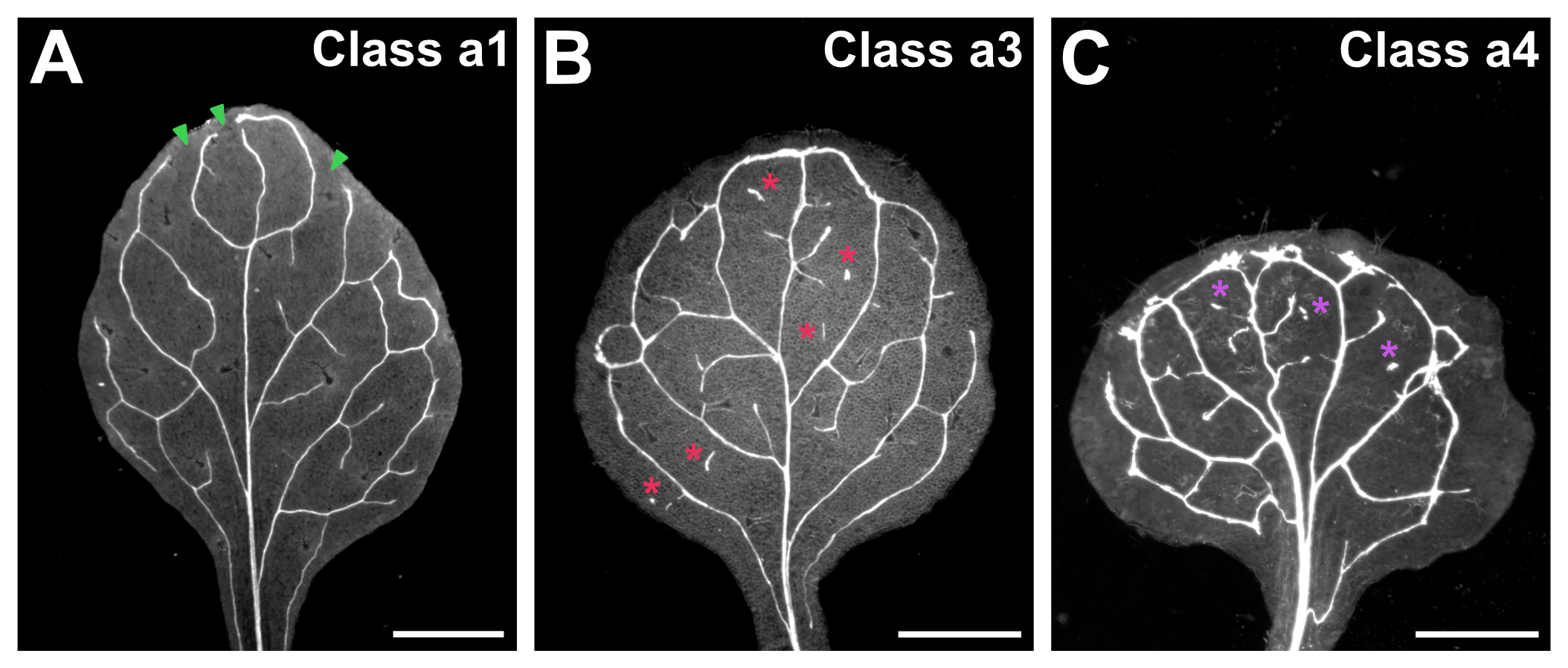

Supplement: S1 Fig — Dark field illumination of mature first leaves illustrating phenotype classes (top right). Class a1: open vein network outline (A); class a3: vein fragments and/or vascular clusters (B); class a4: lobed leaf and vein fragments, and/or vascular clusters (C). Arrowheads: open loops; asterisks: vein fragments and vascular clusters. Bars: 1 mm. (TIFF) [file pbio.3001781.s006.tiff]
