# Supplementary material for: Leaf vein patterning is regulated by the aperture of plasmodesmata intercellular channels
Source: PLoS Biol. 2022 Sep 27;20(9):e3001781. doi: 10.1371/journal.pbio.3001781 (PMC9514613; doi:10.1371/journal.pbio.3001781)
Supplement: Attachment — Submitted filename: Response to Reviewers.pdf [file pbio.3001781.s010.pdf]

Dear editors, Dear reviewers,

Please find attached our revised manuscript “Control of Leaf Vein Patterning by Regulated Plasmodesma Aperture”, and below our point-by-point response to the reviewers’ comments.

To facilitate the identification of the changes we introduced in the original manuscript, we have submitted a revised manuscript that was generated from a Word document in which the “Track Changes” option had been turned on. For simplicity, however, we did not track the addition of new references to the “References” section of the manuscript.

We believe our new text and documentation, including direct evidence that *GNOM* controls permeability of plasmodesmata, have taken all remarks into full account and would like to thank editors and reviewers for their help, time, interest, and patience.

Sincerely,

Enrico Scarpella & Nguyen Manh Linh

***

REVIEWER 1

> The authors investigate the role of plasmodesmatal aperture regulation on leaf venation patterning in Arabidopsis. By using an array of narrow and wide plasmodesmata aperture mutants, combined with a series of ET>>erGFP/YFP enhancer trap drivers, the authors show that plasmodesmatal permeability changes during vein development. Defects in plasmodesmatal aperture influence auxin transport and signalling. Plasmodesmatal aperture depends on the action of the regulator GNOM (GN) and simultaneous inhibition of PD aperture, auxin transport and auxin signalling phenocopies gn mutants. Vein patterning takes place through three GNOM-dependent pathways controlling auxin transport, signalling and plasmodesmata aperture.
>
> **General considerations**
>
> The combined use of the ET>>erGFP/YFP constructs with wide and narrow aperture mutants is a very clever system for this type of investigation. The results are intriguing and shed light on the role of plasmodesmatal permeability on vein patterning. The model reported in Fig.7 is effective in clarifying the findings.
>
> Although I appreciate the thoroughness of the manuscript, it is too long. It would be more effective if shorter and more concise, with better outlined methods.

We thank Reviewer 1 for their positive evaluation of our manuscript. We have striven to shorten the manuscript according to all the reviewers' suggestions, but those efforts have been counteracted by the same reviewers' requests to include additional experiments, along with their description and discussion, and to add more information in the "Materials & Methods". As a result, the total length of the revised manuscript is similar to that of the original manuscript. However, we believe the novelty of our findings requires the rigor and attention to detail Reviewer 1 appreciated. Finally, we would like to point out that the length of our manuscript falls within the word limit for full-length articles recommended by most major journals in biology and that unlike those journals *PLoS Biology* has no such restriction.

Detailed comments on the manuscript

Abstract and introduction

Please consider a more careful choice of wording for the last sentence in both your abstract and introduction sections. Vein patterning may proceed with different mechanisms in other species.

We have now removed any claim that we have identified all the pathways controlling vein patterning in plants.

Vein classification terminology

The terminology used to indicate vein ranks is inconsistent. For example, Fig.1a shows a nice schematic of the terminology used to indicate Arabidopsis vein ranks (midvein, loops and minor veins). However, dotted around in the main text definitions such as "laterals" appear. Please clarify.

Each vein loop results from the amalgamation of a lateral vein with a marginal vein. We have now illustrated that in Fig. 1A and clarified it in its legend.

Materials and methods

Please state what the corresponding controls were in the "chemicals" section.

We have now included the missing information in the "Chemicals" section of the "Materials & Methods".

Very little information is provided regarding the parameters used for the subdivision of phenotypes into classes. It would be useful to have a general description of how classes were sorted.

Description of the phenotype classes, including their defining features, is included in the legend to Figures 1 and 6, and in the new Table S1. In addition, salient features of WT, *cals3-d*, and *gsl8* vein patterns are also described in the “Control of Vein Patterning by Regulated PD Aperture” section of the “Results”. Furthermore, phenotype classes are exemplified in Fig. 1B–E, in the new Figure S1, and in Fig. 6A–F. Finally, we have now added in-panel labels to more easily identify distinguishing features of phenotype classes in Fig. 1B,C and in the new Figure S1. Please also note that in Figure 1 it was necessary to differentiate between class a1 and class a2 and between class a3 and class a5 in order to assess mutant allele strength. However, because that assessment was no longer necessary in Figure 6, where we used a single allele for each mutant, for simplicity we there conflated class a1 with class a2, and class a3 with class a5.

> Regarding the "imaging" section: were the same parameters used for imaging of YFP in all erGFP/YFP samples and thus, are the different panels comparable? Some images seem a bit overexposed, particularly those of young leaves. Was this was done on purpose to compare the samples or should different panels be considered separately instead.

For each ET driver, images of ET>>erGFP/YFP leaves were acquired such that they could be compared with one another. Specifically, we found that expression of both erGFP and YFP is weaker at later stages of leaf development. Furthermore, the numerical aperture of microscope objectives needed to image those later stages (e.g., 0.45 for a 10x objective) is much lower than that of objectives used to image earlier stages of leaf development (e.g., 0.8 for a 20x objective and 1.2 for a 40x objective). Therefore, we first adjusted acquisition parameters (i.e. laser transmission, detector gain, and detector offset) for the oldest leaves (typically 6 DAG) such that signals were saturated only in up to ~1% of the pixels in the acquired images. We then used the same parameters for younger leaves, which led to images in which signals were saturated in >1% of the pixels in the acquired images but ensured that for each ET driver all the images of ET>>erGFP/YFP leaves could be compared to one another. If we had done the opposite — i.e. first adjust acquisition parameters for the youngest leaves (2 DAG) such that signals were saturated only in up to ~1% of the pixels in the acquired images and then use those parameters to image older leaves — signals in the oldest leaves would have been undetectable. We have now clarified this in the “Imaging” section of the “Materials & Methods”.

> *Results*
>
> Fig.1
>
> The classification of phenotypes is quite confusing. For example, classes 0, a2 and a6 in Fig.1 are evidently different; however, I struggle to see the difference between a2 and a5 and I don't know what a1, a3 and a4 look like since there is no representative image in the main text, nor within the supplementals. Adding some more information about what the different

phenotype classes look like, and a corresponding methodology section in the Materials & Methods will help clarify this point.

As mentioned above, description of the phenotype classes, including their defining features, is included in the legends to Figures 1 and 6 and in the new Table S1. In addition, salient features of WT, *cals3-d*, and *gsl8* vein patterns are also described in the “Control of Vein Patterning by Regulated PD Aperture” section of the “Results”. Furthermore, phenotype classes are exemplified in Fig. 1B–E; in the new Figure S1, which includes images of leaves in classes a1, a3, and a4; and in Fig. 6A–F. Finally, we have now added in-panel labels to more easily identify distinguishing features of phenotype classes in Fig. 1B,C and in the new Figure S1. As for the difference between class a2 and class a5, as explained in the legend to Figure 1 and in the new Table S1, leaves in class a5 contain vein fragments and/or vascular clusters, whereas leaves in class a2 contain neither. And representative images for classes a1, a3, and a4 are now provided in the new Figure S1.

Fig.3

Please indicate the site of IAA application.

We have now indicated with an arrowhead the site of IAA application in Fig. 3A,B,E,F.

Fig.4

I couldn't help but notice that the gsl8-chor line was used for panels Y-AC, instead of the gsl8-2 line used throughout the rest of the manuscript. Quite likely they behave similarly, given the classification shown in Fig.1 and the fact that both are strong mutants. Did you also test the gsl8-2;PIN1-YFP line and, if not, what was the motivation for choosing the gsl8-chor line instead.

We found that PIN1::PIN1:YFP is silenced in *gsl8-2*, which is why we analyzed PIN1::PIN1:YFP expression in *gsl8-chor*, whose strength — as illustrated in Fig. 1F — is indeed no different from that of *gsl8-2*.

Fig.5

What is the difference between the DR5rev-nYFPHS and DR5rev-nYFPES reporters? At first glance it seems that the same DR5rev repeats were used for both constructs. I am puzzled by the fact that the same stage of development (4DAG) displays different DR5 patterns (panels 5P and 5R): as an example, there is no strong DR5 activation at the leaf tip in 5R, compared to 5P. Furthermore, it is not clear to me what mechanism could lead to lower and broader DR5 activation in both the DR5rev-nYFPHS;cals3-2d and DR5rev-nYFPHS;gsl8-2 lines.

In DR5rev::nYFP^{HS} (the "H" stands for "Heisler", who generated the construct in Heisler et al., 2005, *Curr. Biol.* 15: 1899–1911 and kindly shared it with us, and the "S" stands for "Sawchuk", who generated the line in the Col-0 background in Sawchuk et al., 2013, *PLoS Genet.* 9: e1003294), the DR5rev promoter drives expression of a fusion between three tandem repeats of the Venus YFP and a fragment of At4g19150, which localizes YFP expression to the nucleus. By contrast, in DR5rev::nYFP^{ES} (the "ES" stands for "Enrico Scarpella", who generated both construct and line in this manuscript), the DR5rev promoter drives expression of the enhanced YFP, which is localized to the nucleoplasm by fusion to three copies of the nuclear localization signal of the Simian Virus 40. We found that the DR5rev::nYFP^{HS} line is more sensitive than the DR5rev::nYFP^{ES} line, conceivably because of the higher quantum efficiency of Venus and the number of Venus repeats. As a result, expression domains of DR5rev::nYFP^{HS} are typically broader than those of DR5rev::nYFP^{ES}. However, we found that YFP expression persists longer in the DR5rev::nYFP^{HS} line. And as a result, leaf tip expression is still detectable in the DR5rev::nYFP^{HS} line at 4 DAG, by which stage it is no longer detectable in the DR5rev::nYFP^{ES} line. We used DR5rev::nYFP^{ES} in *gsl8-2* because we found that DR5rev::nYFP^{HS} is silenced in that mutant.

As for what mechanism could lead to lower levels and broader domains of DR5rev expression in both *cals3-2d* and *gsl8-2*, in the "Discussion" we speculated that near-constitutively narrow PD aperture in *cals3-2d* limits the accumulation in the veins of an auxin-dependent signal produced in nonvascular tissues. In the "Discussion", we also speculated that near-constitutively wide PD aperture in *gsl8-2* depletes veins of an auxin signal accumulated there by diffusion from the nonvascular tissues where it is produced. However, the precise mechanism by which both near-constitutively wide and near-constitutively narrow PD aperture lead to similar DR5rev expression defects remains to be determined.

Fig. 6

> As for Fig. 1, concerns about phenotype classification stand for Fig. 6 too.
> Furthermore, Fig.6g lacks quantification of classes for gn-13;gsl8-2 mutants; my understanding is that, presumably, the distribution would be similar to that of gn-13. Is there a reason why this was not quantified, since at p.17 you say that phenotypes of gn-13;cals3-3d, gn-13;cals3d-2d and gn-13;gsl8-2 are equivalent to those observed for gn-13?

As mentioned above, description of the phenotype classes, including their defining features, is included in the legend to Figures 1 and 6, and in the new Table S1. In addition, salient features of WT, *cals3-d*, and *gsl8* vein patterns are also described in the "Control of Vein Patterning by Regulated PD Aperture" section of the "Results". Furthermore, phenotype classes are exemplified in Fig. 1B–E, in the new Figure S1, and in Fig. 6A–F. Finally, we have now added in-panel labels to more easily identify distinguishing features of phenotype classes in Fig. 1B,C and in the new Figure S1. Please also note that in Figure 1 it was necessary to differentiate between

class a1 and class a2 and between class a3 and class a5 in order to assess allele strength. However, because that assessment was no longer necessary in Figure 6, where we used a single allele for each mutant, for simplicity we there conflated class a1 with class a2 and class a3 with class a5.

As for the data suggesting that *gn-13*;*gsl8-2* leaves are no different from *gn-13* leaves, those data were in the legend to Figure 6; we have now moved them to the “Control of PD-Mediated Vein Patterning by *GN*” section of the “Results”, where they more appropriately belong.

> *Discussion*
>
> The discussion is generally quite thorough and I appreciated the summary model in Fig.7. However, the "Control of PD aperture regulation by GN" section could be expanded, given that this is one of the key points of your work on which the model in Fig.7 is built on. Is there any evidence of GN regulating or interacting with proteins that regulate PD aperture?

To our knowledge, the only published evidence that is consistent with a role for *GN* in the control of PD aperture regulation is the abnormal accumulation of callose in *gn* mutants and BFA-treated WT (Nielsen et al., 2012, *Proc. Natl. Acad. Sci. USA* 109: 11443-11448), and we referred to that evidence in the "Control of PD aperture regulation by *GN*" section of the “Discussion”. However, we have now provided evidence that regulation of PD permeability is defective in *gn* mutants (Fig. 6N–Q and accompanying text in the “Control of PD-Aperture-Dependent Vein Patterning by *GN*” section of the “Results”), and we have now referred to that evidence in the “Control of PD Aperture Regulation by GN” section of the “Discussion”.

> On the same note: at times, various potential explanations of the results are provided but it's not clear which you consider more likely in the context of your model. That is the case for example, on pages 19 and 21. The discussion could be more focused and concise to better support your Fig.7 model.

All the possibilities we propose to account for our results are based on published evidence, are consistent with the model in Figure 7, and are therefore pertinent and worthy of consideration. We do understand that mentioning only one of those possibilities would shorten the “Discussion”, but the choice of which possibility to mention would be arbitrary and therefore — we believe — unjustified.

REVIEWER 2

> This manuscript studies the mechanisms of auxin-induced leaf vein patterning. Through a successful combination of established mutant lines and pharmacological approaches, the authors have found the importance of the movement of auxin or an auxin-dependent signal

through PDs. This could be a missing piece in the GN-dependent vein-patterning pathway, which interacts in a coordinated manner with two other pathways, auxin signalling and auxin polar transport. This has a potential to be accepted to the PLOS Biology, but there are still some concerns that needed to be addressed.

We thank Reviewer 2 for their encouraging assessment of our manuscript.

Major:

Lack of vascular tissue continuity seems to be a consequence of an early defect. At that early stage, GFP moves very freely among all leaf cells suggesting that PDs are not very tightly regulated at such early time point. It would be important to provide more direct evidence that mutant backgrounds used in the study are directly involved in leaf vascular tissue development, that could include:

- assessing expression pattern of Cals3 and GLS8 in the context of leaf vein development

- assessing levels of callose accumulation in the cells of developing vascular tissue

- assessing PD permeability in the mutant backgrounds used as tools for studying PD permeability (cals3, gls8). The authors should include those two mutant lines in the fluorescent protein mobility assay. Future approach could benefit from gauging PDs with bigger proteins like 2xYFP, 3xYFP

We now show abnormal regulation of PD permeability between veins and nonvascular tissues in both *cals3-2d* and *gsl8-2* mutant leaves (Fig. 2Y–AB and accompanying text in the "PD Permeability Changes During Leaf Development" section of the "Results"). In particular, our new results suggest that in *cals3-2d* veins are symplastically isolated already from early stages of leaf development and that in *gsl8-2* veins fail to become symplastically isolated even at late stages of leaf development. We believe we have thus provided the most stringent experimental test among those suggested by Reviewer 2 to support the use of those two classes of mutants to test the hypothesis that the movement of an auxin signal that controls vein patterning and that is not mediated by auxin transporters is mediated by PDs.

The study is missing direct evidence that GNOM controls permeability of PDs (e.g movement of GFP in the gn mutant; callose levels at the WT and mutant's PDs).

We have now provided evidence that regulation of PD permeability is defective in *gn* mutants (Fig. 6N–Q and accompanying text in the "Control of PD-Aperture-Dependent Vein Patterning by *GN*" section of the "Results").

> The authors wrote that strong vascular phenotypes like vascular clustering, may occur in the strong mutant of both pathways: polar auxin transport plus auxin signalling. Both of these pathways are controlled by GNOM. Are such mutants affected in PD permeability?

In Fig. 4G–K, we showed that the auxin transport inhibitor NPA delays the reduction in PD permeability that occurs between veins and nonvascular tissues in normal leaf development. NPA binds PIN proteins and inhibits their activity (Abas et al., 2021, *Proc. Natl. Acad. Sci. USA* 118: e2020857118; Teale et al., 2020, *EMBO J.* 40: e104416). Furthermore, treatment of both WT and mutant backgrounds with NPA phenocopies vein patterning effects of simultaneous mutation in *PIN1*, *3*, *4*, *6*, *7*, and *8*, which in turn provide all the NPA-sensitive vein patterning in the leaf (Verna et al., 2019, *eLife* 8: e51061). We therefore believe the data in Fig. 4G–K suggest that PIN-mediated auxin transport controls PD permeability and have now clarified this in the "Auxin-Transport-Dependent Vein Patterning and Regulated PD Aperture" section of the "Results".

In Fig. 5L–O, we showed that the auxin signaling inhibitor PBA, which phenocopies effects of auxin signaling mutations such as *axr1*, *tir1*;*afb2*, and *mp* (Matthes & Torres-Ruiz, 2016, *Development* 143: 4053–4062; Verna et al., 2019, *eLife* 8: e51061) in both WT and mutant backgrounds, promotes the restriction in PD permeability that occurs between veins and nonvascular tissues in normal leaf development. We therefore believe these data suggest that auxin signaling controls PD permeability and have now clarified this in the "Auxin-Signaling-Dependent Vein Patterning and Regulated PD Aperture" section of the "Results".

> Fig. 3 shows only the images only from before and after the treatment. It's hard to assess how images showing vein loops after the treatment differ from untreated leaf. Please provide images (supplementary, time course) of intermediated stages, clearly illustrating effect of auxin application.

We did that extensively when we first adapted the technique to developing leaves (Sawchuk et al., 2007, *New Phytol.* 176: 560–571), including time-lapse imaging of the development of untreated leaves and of that of leaves to which instead auxin had been applied. We did that again more recently in a detailed protocol (Linh & Scarpella, 2022, *Curr. Protoc.* 2: e349). Sawchuk et al. (2007) was already cited in our manuscript; we have now also cited Linh & Scarpella (2022).

> The difference between Figs 5N and 5O is unclear. It should be quantified in some way.

We have now quantified that difference and reported the results of that quantification in the "Auxin-Signaling-Dependent Vein Patterning and Regulated PD Aperture" section of the "Results" and in the Figure 5 – Source Data 1 file.

Many of the sentences are too long and thus hard to follow. Few examples are:

"To test this prediction, we applied the natural auxin indole-3-acetic acid (IAA) to one side of 3.5-DAG first leaves of E2331 and Q0990, and — because cals3-2d and gsl8 leaves develop more slowly than WT leaves (see below) — of 4.5-DAG first leaves of E2331;cals3-2d and Q0990;gsl8 (Fig. 3A,B,E,F)."

"Moreover, only in ~30% (8/27) of the E2331;cals8-2d leaves in which veins did form in response to IAA application did these veins connect to the midvein: in the remaining ~70% of the responding leaves, the veins whose formation was induced by IAA application ran parallel to the midvein through the leaf petiole (Fig. 3D)."

"Should the auxin-transporter-unmediated movement of an auxin signal that controls vein patterning be mediated by PDs, defects in PD aperture regulation would enhance vein patterning defects induced by auxin transport inhibition."

We have run the "Results", from which those sentences are coming, through ProWritingAid and reduced average sentence length to improve readability. Now the average sentence in the "Results" is no longer than 20 words, which is standard for popular science writing, and the readability level is no higher than Grade 11, i.e. High-School level.

Minor:

It would be easier to follow the information in the figures if all panels were described with genotype and treatment. This could be resolved with the annotation above or below the image itself.

We have now added treatment information to Fig. 3J, Fig. 4A–C,G,I, Fig. 5A,D,G,L,N, Fig. 6H,M, and genotype information to Fig. 4L–AG. Information on genotype, stage (in DAG), and treatment were already in all the other figure panels.

REVIEWER 3

Scarpella and Linh focuses on understanding the molecular pathways involved in vascular development and leaf patterning. The authors have previously addressed the role of auxins in this developmental process and identified GN as a regulatory signalling hub. Here, the authors built on these findings and propose a role for plasmodesmata in this process. There are previous evidences showing Plasmodesmata contribute to auxin distribution mediated by the expression of callose synthases (e.g., GSL8 by Han et al., 2014) and modelling approaches (Mellor et al, 2020). There is also previous papers showing the role of plasmodesmata in vascular development. Based on those it is not surprising that when the authors modify

plasmodesmata aperture (using GSL mutant lines), they find defects in vascular development and auxin transport. The novelty of this paper is in relating this auxin-callose regulatory pathway with the signalling hub GN which acts in leaf vascular development thus my comments below focus on the questions: is there enough evidences to support a GN-dependent pathway regulating callose at plasmodesmata and symplasmic auxin transport? To summarize my conclusions, I think the authors did a lot of work to demonstrate the link between callose synthesis, symplasmic transport, vascular development and auxin but, the link to GN in my opinion is weak and lack mechanistic understanding. There is merit in showing that auxin signalling interacts with callose regulation in the context of leaf patterning but not enough evidence to demonstrate that GN-dependent signalling is directly involved and the effect this has on plasmodesmata structure and function. The authors should moderate their conclusion and interpretation of the experiments provided here.

We thank Reviewer 3 for their constructive feedback on our manuscript. We responded to the reviewer's reservations in the specific comment section below, where those reservations are elaborated by the reviewer. As for whether it is surprising or not that interfering with PD aperture regulation leads to defects in vein patterning and auxin transport, we would like to respectfully suggest that such a surprise is subjective and inconsequential to the advancement of knowledge: to theoretically expect that those defects would result from interference with PD aperture regulation is one thing; it is an entirely different one to show it experimentally, as we did in our manuscript.

Major comments:

1- Abstract claims: 'Therefore, veins are patterned by the coordinated action of three GN-dependent pathways: auxin signaling, polar auxin transport, and movement of an auxin signal through PDs. We have identified all the key vein-patterning pathways in plants' How the authors know that 1) there is no evidence excluding other key-vein patterning pathways. The absence of evidence of not evidence of absence and 2) the results do not necessarily demonstrate the movement of an auxin signal through PDs, they indicate that a signal via PD contribute to auxin distribution. This is not new as per reported by Han et al., 2014 and Mellor et al., 2020 to cite 2 examples.

As for the first point, we have now removed any claim we have identified all the pathways controlling vein patterning in plants. As for the second point, we used the term "auxin signal" as defined in the "Results" section i.e. "auxin or an auxin-dependent signal". In the "Abstract", we have now replaced "auxin signal" with its definition. Finally, as for the novelty of our findings, both Han et al. (2014) and Mellor et al. (2020) — both cited in our manuscript — provided evidence consistent with a role for movement of auxin or an auxin-dependent signal through PDs during *cell elongation* in hypocotyl and root, respectively. By contrast, our manuscript is the first report of the impact of such a movement on the *patterning of an entire tissue system* —

the vascular one. However, in agreement with the manuscript preparation guidelines of major journals, we avoided any claims of priority.

> 2- Related to the above, end of intro indicates ' In the most severe cases, the vascular system of leaves in which those three pathways have been inhibited is no more than a shapeless cluster of vascular cells, suggesting that we have identified all the main pathways in vein patterning' these mutants, inhibitor treatments have many other phenotypes, that could be interacting or even responsible of the leaf vascular patterning, How the authors can read on the leaf vascular patterning alone to get this conclusion without considering these many other defects? Casl3 and gsl8 affects whole plant development (stomata development, root growth, meristem formation, cell plate formation, cytokinesis etc.).

As indicated above, we have now removed any claim we have identified all the pathways controlling vein patterning in plants. However, we never claimed that the interaction between those three pathways only controls vein patterning. And indeed, we had explicitly acknowledged at the end of the "Discussion" that the interaction between those pathways may control other patterning processes — for example, patterning of stomata, which we have now supported with an additional recent reference (Wang et al., 2022, *Nat. Commun.* 13: 7). Notwithstanding the involvement of those three pathways in other processes, we finally provide evidence that regulation of PD permeability between veins and nonvascular tissues is defective in both *cals3-2d* and *gsl8-2* mutant leaves (Fig. 2Y–AB and accompanying text in the "PD Permeability Changes During Leaf Development" section of the "Results"). In particular, our new results suggest that in *cals3-2d* veins are symplastically isolated already from early stages of leaf development and that in *gsl8-2* veins fail to become symplastically isolated even at late stages of leaf development. We believe we have thus experimentally justified the use of those two classes of mutants to test the hypothesis that the movement of an auxin signal that controls vein patterning and that is not mediated by auxin transporters is mediated by PDs.

> 3- Start of the result 'Here we tested the hypothesis that the movement of an auxin signal that controls vein patterning and that is not mediated by auxin transporters is mediated by PDs.' I like this very much. This is the right framing of their hypothesis, which left me wondering why this is oversold/ overinterpreted elsewhere in the paper?

The purpose of the study was indeed that of testing that hypothesis. We have now used the feedback provided by all reviewers to ensure our conclusions do not read as if we were trying to oversell our findings or overinterpret our data.

> 4- Section Control of Vein Patterning by Regulated PD Aperture: The authors should consider using different plasmodesmata mutants to clearly link plasmodesmata to vein formation. So far the results only demonstrate that callose regulation (these mutants are not only affecting PD-callose but also callose in cell plates and sieve pores etc.) affects leaf vascular

development, among the many other phenotypes these mutants have. Vaten et al., Dev cell 2013 already described the effect of cals3 in vascular development thus I am not sure how much here it is new.

To test the hypothesis that the movement of auxin or an auxin-dependent signal that controls vein patterning and is not mediated by auxin transporters occurs through PDs, we selected gain-of-function mutants in *CALS3* and loss-of-function mutants in *GSL8*. Those mutants were selected for two reasons: (1) they are embryo viable; (2) their mechanism of action on PD aperture is known. Other mutants have been reported to affect PD function, but they either lead to embryo lethality or their mechanism of action on PD aperture has yet to be clarified (e.g., Patton et al., 1991, *Mol. Gen. Genet.* 227: 337–347; Kim et al., 2002, *Development* 129: 1261–1272; Yamagishi et al., 2005, *Plant Physiol.* 139: 163–173; Kobayashi et al., 2007, *Plant Cell* 19: 1885–1897; Benitez-Alfonso et al., 2009, *Proc. Natl. Acad. Sci. USA* 106: 3615–3620; Stonebloom et al., 2009, *Proc. Natl. Acad. Sci. USA* 106: 17229–17234; Xu et al., 2012, *Proc. Natl. Acad. Sci. USA* 109: 5098–5103; Burch-Smith & Zambryski, 2010, *Curr. Biol.* 20: 989–993; Stonebloom et al., 2012, *Plant Physiol.* 158: 190–199 — most of which are cited in our manuscript). Furthermore, that our choice of *cals3-d* and *gsl8* mutants to interfere with PD aperture is justified is supported by the many studies that use either of those mutants for the same purpose (e.g., Guseman et al., 2010, *Development* 137: 1731–1741; Vaten et al., 2011, *Dev. Cell* 21: 1144–1155; Daum et al., 2014, *Proc. Natl. Acad. Sci. USA* 111: 14619–14624; Han et al., 2014, *Dev. Cell* 28: 132–146; Wu et al., 2016, *Proc. Natl. Acad. Sci. USA* 113: 11621–11626; Gao et al., 2020, *Curr. Biol.* 30: 1970–1977; Mellor et al., 2020, *Development* 147: dev181669 — most of which are also cited in our manuscript). Finally, the defects in protoxylem and metaxylem differentiation in the vascular cylinder of *cals3-d* roots in Vaten et al. (2011) — also cited in our manuscript — suggest a role for PD aperture in controlling the switch between alternate cell fates within a given tissue. By contrast, our study suggests a role for PD aperture in controlling the patterning of a whole tissue system — a function that involves at least three separate tissue-system-wide processes: tissue formation, maintenance of tissue continuity, and formation and maintenance of tissue connectedness.

5- Note that callose synthesis at plasmodesmata is counteracted by its degradation, thus there is a need to evaluate the expression profile and regulation of beta 1,3 glucanases in this context.

It is conceivable that callose degradation, and not only callose production, has a role in vein patterning. Indeed, at an early stage of our work we analyzed single and double mutants in *PLASMODESMAL-LOCALIZED β-1,3 GLUCANASE 1* (*PDBG1*) and *PDBG2* and found their vein networks to be indistinguishable from those of WT, suggesting no or redundant function of those genes in vein patterning. In the future, it would be interesting to distinguish between those possibilities by creating higher-order *pdbg* mutants — there are at least four and possibly nine *PDBG* genes, according to Benitez-Alfonso et al., 2013, *Dev. Cell* 26: 136–147. The

importance of *PDBG* genes in callose metabolism notwithstanding, dissecting redundant and nonredundant functions of *PDBG* genes in vein patterning would require a whole separate study. Moreover, and most important, such an investigation is clearly outside the scope of our current manuscript.

> 6- the use of chemicals and inhibitors to address auxin role is difficult to interpret specially in the callose mutant phenotypes. Plasmodesmata and callose are very susceptible to changes in the media, including mild osmotic changes. Has this be considered?

We do not believe the use of auxin or inhibitors of auxin transport or signaling is problematic or difficult to interpret. For example, the auxin transport inhibitor NPA binds PIN proteins and inhibits their activity (Abas et al., 2021, *Proc. Natl. Acad. Sci. USA* 118: e2020857118; Teale et al., 2021, *EMBO J.* 40: e104416). Furthermore, treatment of both WT and mutant backgrounds with NPA phenocopies vein patterning effects of simultaneous mutation in *PIN1*, *3*, *4*, *6*, *7*, and *8*, which in turn provide all the NPA-sensitive vein patterning in the leaf (Verna et al., 2019, *eLife* 8: e51061). Likewise, the auxin signaling inhibitor PBA phenocopies effects of auxin signaling mutations such as *axr1*, *tir1*;*afb2*, and *mp* in both WT and mutant backgrounds (Matthes & Torres-Ruiz, 2016, *Development* 143: 4053–4062; Verna et al., 2019, *eLife* 8: e51061). That the responses induced by IAA, NPA, or PBA were specific to the chemical was determined through the use of controls treated with the chemicals' respective solvents, as now indicated in the "Chemicals" section of the "Materials & Methods". In all our experiments, other variables, such as base medium composition and growth conditions, were unchanged and therefore by definition controlled. Finally, we analyzed over 6,000 leaves in our study, and all our observations are highly reproducible, as evidenced by the reproducibility indices reported in the panels of Figures 1–6, in the new Table S1, and in the source data files for Figures 1, 5, and 6. Taken together, all of these considerations suggest that the conclusions we drew from our experiments with IAA, NPA, and PBA are justified.

> 7- Is plasmodesmata regulation affected in gn? Expression of symplasmic reporters in this mutant background could help address this question. Maybe electron microscopy is necessary to visualize plasmodesmata in the mutants.

We have now provided evidence that regulation of PD permeability is defective in *gn* mutants (Fig. 6N–Q and accompanying text in the "Control of PD-Aperture-Dependent Vein Patterning by *GN*" section of the "Results").

> 8- The authors conclude epistatic effects in gn,cals double mutants but gn13 mutants already have a very strong phenotype that would obscure any evaluation of epistatic or additive effects.

Though the *gn* phenotype is indeed very abnormal, it still leaves room for additive and even super-additive effects. For example, mutation in *GN-LIKE 1* / *ENDOPLASMIC RETICULUM MORPHOLOGY 1* (*GNL1* hereafter) — which incidentally leads to much milder defects than those induced by the *cals3-d* or *gsl8* mutation — leads to completely penetrant gametophytic lethality in the *gn* background (Richter et al., 2007, *Nature* 448: 488–492; Nakano et al., 2009, *Plant Cell* 21: 3672–3685). The gametophytic lethality of the *gn*;*gnl1* double mutant is a case of synthetic enhancement that clearly shows that the *gn* phenotype is not a terminal phenotype and that therefore our interpretation of epistasis of the *gn* phenotype to the *cals3-d* or *gsl8* phenotype is justified. Similar considerations have led to the conclusion that the *gn* phenotype is epistatic, for example, to the *mp*, *axr1*, and *pin1*;*3*;*4*;*6*;*7*;*8* phenotypes (Mayer et al., 1993, *Development* 117: 149–162; Verna et al., 2019, *eLife* 8: e51061).

> 9- The discussion is extensive (10 pages) and could be easily condensed. The last section is unnecessary and distracting.

The number of pages is of course relative: we used a wide typeface, abundant margins, and generous line spacing — all good typographic practices to improve readability that, however, result in longer text spanning more pages than single-spaced 12-point Times New Roman and 1-inch margins. Moreover, the length of our manuscript falls within the word limit for full-length articles recommended by most major journals in biology, and unlike those journals *PLoS Biology* has no such restriction. Finally, the "Discussion" is approximately two-thirds of the length of the "Results", which is standard in the field (e.g., Hopkins & Dudley-Evans, 1988, *Eng. Specif. Purp.* 7: 113–121; Peacock, 2002, *System* 30: 479–497; Kanoksilapatham, 2005, *Eng. Specif. Purp.* 24: 269–292). We do agree the "Discussion" is thorough, but we believe that thoroughness is necessary to account for how we drew conclusions from specific results, to explain how our conclusions are consistent with previous research, to offer alternative interpretations, and to expose limitations of our study. Nevertheless, as suggested by Reviewer 3, we have now deleted the last paragraph of the "Discussion". We have also moved the second-last paragraph of the "Discussion" from its own separate section ("Beyond Vein Patterning") to the end of the previous section ("A Diffusion–Transport-Based Vein-Patterning Mechanism"). As a result, the section "Beyond Vein Patterning" has now disappeared from the "Discussion".

> 10- Figure legends, the structure of the legends is very confusing to understand. Title are the author's conclusions and there is no detail description of what is shown and how the experiment was performed, instead there is a list of abbreviators, colour grades and objects. I would prefer if the author take the time to describe the experiment and the results shown in each panel.

In agreement with manuscript preparation guidelines of major journals, we believe figure legends should contain neither results nor methods, to which separate manuscript sections are dedicated; rather, figure captions should contain legends to abbreviations and look-up-tables,

as well as information on statistics. In further agreement with manuscript preparation guidelines of major journals, we aligned the titles of the figures legends with the titles of the “Results” sections the figures are supposed to support. Finally, the titles of our figure legends (and of the corresponding “Results” sections for that matter) are descriptive, rather than declarative; as such, they do not imply conclusions.

REVIEWER 4

> This manuscript reports how movement of an auxin signal through plasmodesmata is essential for correct vein formation within the leaf. The study presents a detailed analysis of this process, clearly describing hypotheses and carefully characterising the vein patterns in numerous relevant mutants. Given vein formation is a popular research area, with a long history, the idea that this could be a key refinement of the 'Canalisation hypothesis' is an exciting prospect. Furthermore, auxin diffusion through plasmodesmata has received much interest recently, with several highly cited papers showing this to be an important process in other organs in the last few years. I would therefore expect this manuscript to be of wide and long-standing interest.

We thank Reviewer 4 for their positive evaluation of our manuscript.

> Clearly a huge amount of experimental work has gone into the manuscript, however, I felt in places the relevance of this work in the field, and mechanistic insights could be improved. The work clearly is a key refinement of the Canalisation hypothesis, and yet discussions of the coupling between auxin concentration, and auxin-regulation of PIN transport and plasmodesmatal permeability was delayed until late in the discussion. I have a number of suggestions that I feel would improve this, and generally improve the readability of the manuscript:
>
> 1. In the introduction, I was left wondering (i) whether it was previously known that plasmodesmata play a role in vein formation, and (ii) whether there was previous evidence that plasmodesmata are regulated by GNOM. You write "here we ask whether movement of auxin or an auxin-dependent signal through PDs is one of the missing GN-dependent vein-patterning pathways", but I feel more details are needed as to what was known about this before. It is written of p8 "Consistent with previous observations (Kim et al.)," however, details of Kim et al are not in the introduction. Were these previous observations part of your motivation for suggesting your hypothesis? It would be good to clarify at the outset what was already known.

Before our study, it was unknown whether PDs had any role in vein patterning and whether *GN* controlled any aspect of PD development or function. As naïve as it may sound, our sole motivation for testing the hypothesis that movement of auxin or an auxin-dependent signal

through PDs is one of the missing *GN*-dependent vein-patterning pathways was that experimental evidence (reviewed in Paterlini, 2020, *Biol. Open* 9: 1–11 and Band, 2021, *New Phytol.* 231: 1686–1692, both of which are cited in the “Introduction”) suggested that auxin could move through PDs. As for the reference to Kim et al. (2005), in that paper the Zambryski lab showed that the PD permeability of the cells surrounding the shoot apical meristem decreases during embryogenesis. Though those findings are consistent with our own, the findings by the Zambryski lab had no impact on our motivation to test the hypothesis that movement of auxin or an auxin-dependent signal through PDs is one of the missing *GN*-dependent vein-patterning pathways. As such, we believe it would be distracting to include those findings by the Zambryski lab in the “Introduction”, which instead summarizes all that was known at the outset of our study and that motivated us to pursue our investigation.

> 2. The introduction also briefly mentions that auxin signalling has been shown to play a role in vein formation, however, given these findings are built on in this manuscript, again I felt more details are needed. On p15, you write "auxin-signaling-unmediated movement of an auxin signal" which seems to suggest that it is thought that the auxin signalling is acting through auxin movement. I suggest clarifying this in the introduction.

We have now summarized in the “Introduction” the evidence suggesting that the residual vein patterning activity in auxin-transport-inhibited leaves depends on movement of auxin or an auxin-dependent signal and that such movement of auxin or an auxin-dependent signal with vein patterning function depends, at least in part, on auxin signal transduction.

> 3. On p5, you write "As calsP-d, gslR mutants formed networks" Please rephrase, as at first I thought you were talking about the double mutant.

We have rephrased to remove ambiguity and added a “Notation” section to the “Materials & Methods” to explain the system of symbols we use throughout the manuscript, including the notation for double mutants.

> 4. On p9, you write "To test this prediction, we applied the natural auxin indole-S-acetic acid (IAA) to one side of 3.5-DAG first leaves of E2331" and Q0990" Presumably these are the control lines for the subsequently stated mutants - could this be clearer.

We have rephrased to clarify that indeed E2331 and Q0990 are the respective controls for E2331;*cals3-2d* and Q0990;*gsl8-2*.

> 5. On p11, "Should the auxin-transporter-unmediated movement of an auxin signal that controls vein patterning be mediated by PDs," It would be good to be clearer here as to what is known and what the hypothesis is - the text seems to suggest that some sort of alternative auxin movement has already been established.

We have now summarized in the “Introduction” the evidence suggesting that the residual vein patterning activity in auxin-transport-inhibited leaves depends on movement of auxin or an auxin-dependent signal; that such movement of auxin or an auxin-dependent signal with vein patterning function depends, at least in part, on auxin signal transduction; and that vein patterning activity exists which depends on movement of auxin or an auxin-dependent signal but is independent of auxin transport and signaling.

> 6. The phrase "auxin-transporter-unmediated movement" is somewhat clumsy, and is used in several places - do you simply mean passive movement? This leads on to phrases such as "Should the residual, auxin-transporter- and auxin-signaling-unmediated movement of an auxin signal that controls vein patterning be mediated by PDs" which is somewhat hard to follow! Please rephrase.

We have now rephrased all those cumbersome expressions.

> 7. I also disagree that the phrases involving "PD mediate auxin movement" is accurate - the verb mediate tends to be used for indirect influences, and so is perhaps not suitable to describe how auxin passively moves through the plasmodesmata. I would have said that rather the PD enable passive auxin movement.

We have now replaced “mediate” with “enable”, which is indeed more appropriate.

> 8. p13 describes the PIN1 distribution, could you clarify whether PIN1 exhibits a polar localisation within the cells.

We have now clarified that PIN1 localization remains polar in mutants defective in PD aperture regulation.

> 9. Discussion, p18 "we have identified all the main pathways that control vein patterning." Presumably 'we' is meaning the community, rather than the authors of this paper - may be wise to rephrase!

We have now removed from the manuscript ambiguous claims such as that referred to by Reviewer 4.

> 10. Discussion, p22, That auxin itself is known to regulate plasmodesmatal permeability is a key mechanistic insight that has major implications for this study. I suggest that this point is made earlier. One possibility is that auxin itself is controlling the plasmodesmata dynamics during normal leaf development - I felt this point could be made more explicitly. Previous studies have shown that plasmodesmatal permeability is reduced by auxin however, which

seems counterintuitive given the results presented here show high plasmodesmatal permeability within the veins - perhaps this could be discussed.

The reported effects of auxin on PD permeability may indeed appear confounding. On the one hand, our results suggest that auxin increases PD permeability between veins and nonvascular tissues during leaf development. On the other hand, such a conclusion seems to be inconsistent with the observation that auxin reduces PD permeability during development of lateral roots and bending of mature hypocotyls (Han et al., 2014, D*ev. Cell* 28: 132–146; Sager et al., 2020, *Nat. Commun.* 11: 364) or that auxin has no effect on PD permeability in mature leaves and root tips (Gao et al., 2020, *Curr. Biol.* 30: 1970–1977; Rutschow et al., 2011, *Plant Physiol.* 155: 1817–1826). However, such seemingly inconsistent observations may simply reflect organ (leaf vs. root and hypocotyl) or developmental stage (mature vs. developing) specific responses. Indeed, auxin application only induces vein formation in developing leaves and fails to do so in mature leaves or in hypocotyls and roots of Arabidopsis (Scarpella et al., 2006, *Genes Dev.* 20: 1015–1027; Sauer et al., 2006, *Genes Dev* 20: 2902–2911). We have now explicitly suggested that auxin may control PD permeability during normal leaf development in the “Auxin, Regulated PD Aperture, and Vein Patterning” section of the “Discussion” and provided there an account of how such a conclusion may be reconciled with seemingly inconsistent, already available ones.

11. Discussion, p22. I believe that Han et al also showed auxin-regulation of plasmodesmatal permeability, focussing on gsl8, and showed this regulation to be through the auxin signalling pathway.

We had indeed mentioned that our findings are consistent with the inability — reported by Han et al. (2014) — of *gsl8* plants to restrict expression domains and maintain high expression levels of auxin response reporters in the hypocotyl. However, the finding in Han et al. (2014) that auxin signaling induces *GSL8* expression cannot account for our observation that auxin signaling delays the reduction in PD permeability between veins and surrounding nonvascular tissues that occurs during normal leaf development. Induction of *GSL8* expression by auxin signaling is indeed expected to lead to narrower — not wider — PD aperture and thus lower — not higher — PD permeability. In the closing paragraph of the the “Auxin, Regulated PD Aperture, and Vein Patterning” section of the “Discussion”, we have now suggested how the seemingly contradicting findings in Han et al. (2014) and our study may be reconciled.

12. The discussion section "A Diffusion-Transport-Based Vein-Patterning Mechanism" provided clear insights into that plasmodematal auxin diffusion together with auxin regulation of plasmodesmatal permeability could couple to the canalisation mechanism involving PINs to fully explain vein formation. However, I felt some of these ideas could be introduced early. For instance, that it's established that auxin can regulate plasmodesmatal permeability seems to be key to the results described on p9-10. That coupling with auxin-mediated PIN regulation is

> also thought to be important for vein formation could help understanding of the results on p11-14. Furthermore, an earlier section of the discussion "Regulation of PD Permeability During Leaf Development" doesn't mention auxin regulation, and concludes "the mechanism by which changes in PD permeability are brought about during leaf development is inconsequential to the conclusions we derive from such changes." As mentioned above, I felt that introducing these mechanistic insights earlier and more explicitly would make the paper stronger.

First, as explained above in our response to point 10, whereas an effect of auxin on PD permeability had indeed already been observed during bending of mature hypocotyls and development of lateral roots (Han et al., 2014, D*ev. Cell* 28: 132–146; Sager et al., 2020, *Nat. Commun.* 11: 364), no effect had been observed in root tips and mature leaves (Gao et al., 2020, *Curr. Biol.* 30: 1970–1977; Rutschow et al., 2011, *Plant Physiol.* 155: 1817–1826). Furthermore — unlike developing leaves — hypocotyls, roots, and mature leaves fail to respond to auxin application by forming new veins (Scarpella et al., 2006, *Genes Dev.* 20: 1015–1027; Sauer et al., 2006, *Genes Dev* 20: 2902–2911). Therefore, we believe introducing that information in the "Auxin-Induced Vein Formation and PD Aperture Regulation" section of the "Results" — as if it were the premise to or the motivation for our investigation of the effects of auxin on PD permeability during leaf development — would be misleading and confusing. Instead, we find it most appropriate to refer to that information in the "Auxin, Regulated PD Aperture, and Vein Patterning" section of the "Discussion", where we note that the effect of auxin on PD permeability that had been observed in hypocotyls and lateral roots is opposite to that which we observed in developing leaves, and we provide an account to reconcile those two seemingly conflicting observations.

We agree that the "A Diffusion-Transport-Based Vein-Patterning Mechanism" section of the "Discussion" helps understand our findings, including those in the "Auxin-Transport-Dependent Vein Patterning and Regulated PD Aperture" section of the "Results". However, we also believe that providing an account of the findings is precisely the scope of the "Discussion" and not of the "Results". Therefore, we believe the discussion of the requirement for a coupling mechanism between PD aperture regulation, on the one hand, and auxin signaling and transport, on the other, really belongs to the "Discussion".

Finally, the phrase "the mechanism by which changes in PD permeability are brought about during leaf development is inconsequential to the conclusions we derive from such changes" does not claim or imply that the *signals* — e.g., auxin — that control such mechanism are inconsequential. Instead, the phrase refers to the observation — made in the sentences preceding that phrase — that many of the mechanisms reported in literature to account for changes in PD aperture are compatible with the changes in PD permeability we observed during leaf development and that therefore how symplastic connection between vein cells and their

isolation from surrounding nonvascular tissues are brought about during leaf development remains to be understood.
